# Refugee women’s and providers’ perceptions of person-centered maternity care: a qualitative study in two refugee camps in Chad

**DOI:** 10.1186/s12884-024-06424-z

**Published:** 2024-04-01

**Authors:** Alexis Ngarmbatedjimal, Mahamat Abdelaziz, Vincent de Paul Allambademel, Aminata Diarra, Valentin Djerambete, Thérèse Kodjimadje, Samy Luketa, Robert Madjigoto, Yodé Miangotar, Alladoum Ndingayande, Salomon Tamira, Theodora Varelis, Katchebe Vourbane, Sara E. Casey

**Affiliations:** 1 Laboratoire de Sociologie, d’Anthropologie et des Etudes Africaines (LASA), Department of Sociology, College of Humanities and Social Sciences, University of N’Djamena, BP 1117, N’Djaména, Chad; 2 RAISE Initiative, Heilbrunn Department of Population and Family Health, Mailman School of Public Health, Columbia University, 60 Haven Ave, New York, NY 10032 USA; 3 International Rescue Committee Chad, BP 5208, N’Djaména, Chad

**Keywords:** Respectful maternity care, Sexual and reproductive health and rights, Refugees, Chad, Sudan

## Abstract

**Background:**

Globally, mistreatment of women during labor and delivery is a common human rights violation. Person-centered maternity care (PCMC), a critical component of quality of care, is respectful and responsive to an individual’s needs and preferences. Factors related to poor PCMC are often exacerbated in humanitarian settings.

**Methods:**

We conducted a qualitative study to understand Sudanese refugee women’s experiences, including their perceptions of quality of care, during labor and delivery at the maternities in two refugee camps in eastern Chad, as well as maternity health workers’ perceptions of PCMC and how they could be better supported to provide this. In-depth interviews were conducted individually with 22 women who delivered in the camp maternities and five trained midwives working in the two maternities; and in six dyads with a total of 11 Sudanese refugee traditional birth attendants and one assistant midwife. In addition, facility assessments were conducted at each maternity to determine their capacity to provide PCMC.

**Results:**

Overall, women reported positive experiences in the camp maternities during labor and delivery. Providers overwhelmingly defined respectful care as patient-centered and respect as being something fundamental to their role as health workers. While very few reported incidents of disrespect between providers and patients in the maternity, resource constraints, including overwork of the providers and overcrowding, resulted in some women feeling neglected.

**Conclusions:**

Despite providers’ commitment to offering person-centered care and women’s generally positive experiences in this study, one of few that explored PCMC in a refugee camp, conflict and displacement exacerbates the conditions that contribute to mistreatment during labor and delivery. Good PCMC requires organizational emphasis and support, including adequate working conditions and ensuring suitable resources so health workers can effectively perform.

**Supplementary Information:**

The online version contains supplementary material available at 10.1186/s12884-024-06424-z.

## Background

In 2020, a maternal death occurred nearly every two minutes, 70% of which occurred in sub-Saharan Africa [1]. Although the majority of these deaths are preventable, weak health systems, poor quality of care, lack of resources and skilled providers, and limited access to emergency obstetric and newborn care (EmONC) contribute to this high maternal mortality. Conflict and fragility further exacerbate these issues, by disrupting the health system and the continuum of care [2, 3]. Maternal mortality ratios are twice as high in countries affected by armed conflict as in stable ones [4].

Globally, women often experience human rights violations through acts of obstetric violence or mistreatment during labor and delivery, which act as a deterrent to care-seeking [5–7]. These may be heightened in humanitarian settings where women face increased risks of sexual violence, unintended pregnancies, and unsafe abortions which, along with a disrupted health system and insecurity, contribute to increased maternal mortality and morbidity [5, 8]. In a humanitarian crisis, one of the primary goals is to re-establish or strengthen the disrupted health system to provide emergency and life-saving care, including EmONC; however, quality of care may not be prioritized [9].

Person-centered maternity care (PCMC), a critical component of quality of care, may be even more important in a humanitarian setting given the multiple vulnerabilities women in crises face. PCMC is defined as care that is respectful, responsive to an individual woman's needs, preferences and wants when making clinical decisions [10]. PCMC emphasizes dimensions of care including communication, confidentiality, respect and dignity, and social support that are components of the World Health Organization’s (WHO) quality of care framework for maternal and child health [11, 12]. The universal rights of childbearing women in the Respectful Maternity Care Charter and the WHO statement on the prevention and elimination of disrespect and abuse during facility-based childbirth recognize the critical importance of PCMC [13, 14]. The ability of a health facility to provide a welcoming environment and good quality care is considered in women’s decision-making about where to deliver [6]. PCMC has been shown to be a vital factor in reducing maternal mortality [15, 16].

Existing research has focused primarily on identifying the gaps in PCMC in stable low-resource settings, but little evidence exists on PCMC in humanitarian settings [17]. Violations of PCMC in these settings are heightened through insufficient information, the absence of privacy and consent, denial or delay of care, neglect and abandonment, language barriers and shortage of resources including qualified healthcare providers, all of which may be exacerbated in a humanitarian crisis [17–19].

### Context

As a result of the Darfur, Sudan crisis in 2003, Chad hosted nearly 400,000 Sudanese refugees, the majority of them living in refugee camps in the Eastern part of the country in Wadi-Fira, Ouaddai, Sila, and Ennedi-Est provinces [20]. Despite their presence for over a decade, these refugee camps have high humanitarian needs, and have received over 400,000 new arrivals between April and August 2023 as a result of increased violence in Sudan [21]. The International Rescue Committee (IRC) has supported the health centers in Mile and Kounoungou camps in Wadi-Fira, where this study was conducted, since 2017 with primary health care including sexual and reproductive health services. At the time of this study (before the 2023 influx), Mile and Kounoungou housed 52,135 Sudanese refugees, the majority of whom had lived there for over a decade [22, 23]. Most refugee women in Chad attend antenatal care, with 75% completing four or more visits [24]. Skilled birth attendance is also common (97%) compared to 21.8% of Chadian women in Wadi-Fira [24, 25].

From 2020-2023, IRC in partnership with the Ministry of Health (MOH) and the Association Tchadienne pour le Bien-Etre Familial, implemented the *Protection, Gender and Health* (ProGeSan) program to improve social empowerment of women through better access to maternal, newborn, infant, adolescent and sexual and reproductive health services and response to gender-based violence. ProGeSan was implemented in four refugee camps, including Mile and Kounoungou, in Wadi Fira province and three districts in Guera province. IRC is the main health provider for the two camps, each of which has one health center offering free health care. The camp in Kounoungou has grown to encompass an MOH-run health center that receives support from CARE, including for one midwife. In addition, the MOH District Hospital in Guéreda provides comprehensive EmONC, including cesarean sections. IRC’s ProGeSan program supports the maternity at the camp health center in Mile, and the maternity at the MOH health center serving Kounoungou with antenatal (ANC) and safe delivery care including most basic EmONC signal functions^1^, post-abortion care and contraceptive services. This support includes salary support or compensation for two (Kounoungou) or three (Mile) Chadian midwives and six traditional birth attendants (TBAs) recruited from the refugee population (plus one assistant midwife) in each maternity, and essential supplies and equipment. IRC has facilitated trainings for providers on basic EmONC, prevention of mother to child transmission of HIV, focused ANC, contraception, post-abortion care, care for survivors of sexual assault and adolescent SRH. In addition, IRC and MOH supervisors provide joint supportive supervision, while IRC supervisors provide regular mentoring and coaching to help providers maintain competency. The Chadian midwives have overall responsibility for management of the maternities. The refugee TBAs support the midwives by greeting the patients, taking their vitals and monitoring them in labor. They assist the midwives with delivery care and active management of the third stage of labor but do not give medication.

In collaboration with IRC, the Reproductive Health Access, Information and Services in Emergencies (RAISE) Initiative at Columbia University and the University of N’Djamena conducted a mixed-methods study to understand Sudanese refugee women’s experiences during labor and delivery and their perceptions of quality of care at the maternities in Mile and Kounoungou camps, as well as maternity health workers’ perceptions of PCMC and how they could be better supported to provide this.

## Methods

We conducted a qualitative cross-sectional study at the maternities serving Mile and Kounoungou refugee camps, one of which is managed by IRC (Mile) and one that is managed by MOH with support from IRC (Kounoungou). In-depth interviews were conducted individually with five trained midwives working in the two maternities, and in six dyads with Sudanese refugee traditional birth attendants and one assistant midwife (referred to as refugee providers in this manuscript for clarity and brevity).

In-depth interviews were conducted individually with 22 women (11 per camp) who gave birth in the camp maternities in the six to ten weeks previously. During the 3 months before data collection, midwives explained the study using an information sheet to women prior to discharge after giving birth at the health center, and asked if they would be willing to meet with an interviewer. Women’s decisions were indicated in the register. The research team then extracted names, delivery date, age, parity, Zone and Block numbers for women who agreed to be contacted. Respondents were purposively selected from these lists to ensure a range of age, parity and Zone within in the camp.

IRC recruited three female interviewers who had secondary school education and prior experience conducting qualitative interviews, were from the local community and spoke French and local Arabic. They participated in a three-day training facilitated by members of the research team which covered the concepts of respectful maternity care and interviewing techniques such as probing. Interviewers reviewed interview guides in French, and agreed amongst themselves how best to translate the questions into local Arabic. Interviewers then practiced administering the guide to one another to gain additional familiarity with the questions and subject matter. The semi-structured interview guides were adapted from previously used tools, and covered women’s description of what happened at the health facility on the day of their delivery, communication with the providers, their definitions of disrespectful attitudes or behaviors by a health worker and of good or bad quality health services, and whether they had themselves experienced, witnessed or heard about disrespect and abuse experienced by other women (Additional file 1). Interview guides with the providers included their perception of their work and respect in the workplace, how they define respectful care and practice, what factors influence the provision of respectful maternity care and their observations of mistreatment during childbirth ever in their career.

Community health workers who conduct home visits in the camps accompanied the interviewers to selected women’s homes. When the respondent was not available, the researchers returned later to find her, or if necessary, selected a new respondent from the same or nearby Zone of a similar age and parity to replace her. Interviews were conducted in Arabic with women and refugee providers and in French with the midwives according to the respondent’s preference. A female Chadian Master of Sociology student who helped facilitate the interviewer training, co-author (TK), interviewed the Chadian midwives. All interviews were audio-recorded with the respondent’s consent. Interviews with women were conducted at her home or in a nearby private location, and with providers in a private room at the health facility. Interviews took 25-60 minutes, and were conducted 28 June – 1 July 2022.

Facility assessments were conducted at each maternity to determine their capacity to provide PCMC. The assessments consisted of an inventory of supplies and equipment, as well as brief interviews with midwives and refugee providers in the maternity centers at the time of the assessment. Researchers also took note of the number of beds, as well as the cleanliness and privacy of the labor and delivery rooms and patient bathrooms.

### Analysis

All interviews were transcribed and translated into French if needed by local transcription assistants. French transcripts were then reviewed by bilingual members of the research team to ensure translations were accurate. US and Chadian researchers used an inductive approach to collaboratively create codebooks through an iterative process. Separate codebooks for interviews with women and with providers were then uploaded into Nvivo (version 12). The researchers individually coded one transcript, then met to discuss the codes applied and come to agreement on how to use the codes. Transcripts were then double coded line by line, with the researchers meeting to review the coding after completing two or three transcripts. Interviews with three women and one refugee provider dyad were coded by an individual researcher at the end of the process. Coding queries were generated and thematic analysis was conducted to identify major themes that arose in the interviews [26]. These themes were then interpreted in the context of the study and the domains of PCMC [12] (Additional file 2). Throughout the analysis, the research team reflected on assumptions and preconceptions regarding what constitutes disrespectful care.

### Ethical considerations

Ethical approvals were obtained from Columbia University's Institutional Review Board and the Direction de la Recherche et de l’Innovation, Direction Generale Technique de l’Enseignement Superieur, de la Recherche et de l’Innovation in Chad. The interviewer read an information sheet to potential respondents with an explanation of the study purpose and procedures, potential risks, confidentiality and that participation was voluntary. Respondents provided verbal consent for the interview and its audio-recording. All interviews were held in private spaces; names of respondents and any identifiable information were redacted from written transcripts identified with code numbers that were not linked to participant names. The lists used to identify women were destroyed upon completion of the interviews. Transcripts and recordings of interviews were only accessible to the research team.

## Results

Interviews were completed with 22 women who had delivered at one of the two health centers within the camps in the 6-10 weeks before data collection (Table 1). Although our initial list included two 17 year olds, neither was available for an interview, but ten women were youth aged 18-24 years old. Half of the women had four or more births, while one woman was interviewed about her first birth. The majority, 13 women, reported their first birth was before age 20, and most had at least some formal schooling.

**Table 1:** Demographics of post-partum women interviewed

	**Kounoungou (*n*=11)**	**Mile (*n*=11)**
**Age**		
18-24 years	4	6
25-40 years	7	5
**Number of births**		
1-3	4	6
4 or more	7	5
**Age at first birth**^a^		
15-19 years	6	7
20-25 years	4	4
**Years of schooling completed**		
None	3	0
At least some primary school	4	5
At least some secondary school	4	6

^a^One woman in Kounoungou was missing data

Interviews with maternity staff were completed with five Chadian midwives and 12 refugee providers (Table 2). Refugee providers were Sudanese refugees, 11 of whom were traditional birth attendants and one who had completed assistant midwife training. Most of the TBAs reported some kind of training received in Chad or Sudan. Midwives reported an average of five years of experience, while the refugee providers had an average of 14 years of experience.

**Table 2:** Maternity health workers interviewed

	**Kounoungou (*n*=8)**	**Mile (*n*=9)**	**Interview Type**
Trained Midwives (Chadian)	2	3	Individual
Refugee Providers (Sudanese)	6	6	Dyad

Results were organized by domains of PCMC: dignity and supportive care, communication and autonomy, privacy and confidentiality, social support and health facility environment. Women in this study generally used the term “midwife” to refer to both the Chadian midwives and the refugee providers. Therefore, we have only specified Chadian midwife or refugee provider in the results when the women clearly identified to whom they were referring. We found no systematic differences by camp or by age, so results are reported for all participants.

Overall, women reported positive experiences in the maternities during labor and delivery, and many described mutual respect between providers and patients. Their descriptions of interactions with the providers were far more positive than negative. Providers generally highlighted their satisfaction with their work, and the efforts they made to provide respectful care to women. They largely defined respectful care as patient-centered and respect as being something fundamental to their role as health workers. Across respondents, very few reported incidents of disrespect between providers and patients in the maternity, many of which were linked to overwork of the health workers or disrespect initiated by patients towards the providers. No respondents reported experiencing or observing incidents of physical violence in the maternity.

## Dignity and supportive care

Women overwhelmingly reported being welcomed and treated well at the maternity. They described feeling supported and encouraged by the providers during labor and delivery. Most women said they felt comfortable, as if they were in good hands. Even when the interviewers probed further, reminding women of the confidentiality of the interviews, most reiterated that the midwives and refugee providers treated them well from the moment they arrived. Several specifically commented on the providers’ good quality care, strong teamwork, and pleasant manner and tone of voice.

*With the midwives, I liked their welcome, their explanation on the use of medication and their respect for patients. (Woman, 20 years old, Kounoungou)*

*I appreciated their [the midwives’] help. And especially their moral and physical support during the contractions until the birth of the baby... I felt that they really have a great humanitarianism towards people, and especially a lot of compassion for pregnant women. (Woman, 31 years old, Kounoungou)*

*She [the midwife] gave me consideration, made me feel important and she took good care of me. I didn't see any bad behavior… I have good feelings towards her … Yes I thought she behaved well with me, and with my child too. (Woman, 28 years old, Mile)*

Providers discussed the importance of providing patient-centered care which they described as maintaining a good heart, patience and courage when working with the patients, even if patients were sometimes disrespectful when in pain and/or discomfort during labor and delivery.

*Any woman who comes must be give good respectful care. When you respect someone, the person respects you too. And we must not insult a woman or a pregnant woman, we must always them. (Chadian Midwife)*

The most common negative experience women reported was being left unattended for periods of time while at the health center. A few women arrived at the maternity in labor, but were not seen in a timely manner due to overcrowding, and felt they were forgotten. It is unclear whether the staff in these examples explained why the women were not being attended to immediately, but ultimately, the women viewed this treatment as unacceptable.

*What's more, the supervision is mediocre, and you're left alone. They told us to yell when the baby is coming and we’ll come back to deliver the baby. That's why we say we’ve had a hard time. (Woman, 27 years old, Kounoungou)*

A few women reported instances of disrespect where they themselves were scolded by providers or witnessed another patient being scolded. When asked to expand on how they were mistreated, women described judging tones, disrespectful comments, and simply disregarding or not believing a patient's concerns. Even women who reported having positive birthing experiences themselves shared the opinion that some providers can be on occasion disrespectful towards patients. Some incidents of scolding occurred during ANC, particularly when the woman was not given medicines she was expecting. One woman acknowledged the power imbalance between health workers and patients.

*I never [heard of mistreatment by the midwives]! But they can scold pregnant women who do not come for [antenatal] visits and come in a critical state and want a quick intervention. They say, ‘Why don't you respect the instructions we give you? Why don't you follow up at the center with the midwives?’ (Woman, 23 years old, Mile)*

*We're afraid of them because they have all the power; we have to respect that. (Women, 40 years old, Kounoungou)*

One young woman reported that because of a previous scolding by a maternity provider, that she did not think she would help her deliver her baby if there were no other providers present and reported worrying that her baby would be in danger if she had no help.

*No, I didn't think [the midwife would help me], how is someone who scolds you going to help you give birth? (Woman, 18 years old, Kounoungou)*

Although providers reported mutual respect between provider and patient most of the time, some refugee providers described some instances of midwives being verbally disrespectful towards patients. Both women and providers mentioned this happened when the patient was disrespectful to the provider first, thus prompting the provider to respond similarly, or when the midwives were overworked.

*No, it [respect shown by providers] is not all the time. Sometimes the patients irritate the staff, then they [staff] respond with bad behavior (Sudanese provider)*

*The midwives encounter some patients [during ANC] that don't respect them or complain too much and ask for too much medication… so they [the midwives] get angry and scold the patients. (Woman, 20 years old, Mile)*

The refugee providers also said the midwives who behaved particularly badly no longer worked at this facility. However, they noted that women who had negative experiences were less likely to return to the maternity for care, potentially endangering their health. One midwife described a need for IRC refresher trainings with all maternity health workers to reinforce the importance of respectful care.

## Communication and autonomy

Overall, women reported generally good communication with providers, with only a few exceptions. They described the providers advising them during ANC to arrive earlier at the maternity in labor rather than later, instructing them on the purpose of each medication and how to store and take them and providing guidance on breastfeeding, nutrition and proper hygiene.

*The [Chadian] midwives don't give you medicine without explaining how to use them. They tell us to take such and such a number of tablets per day and per hour. (Woman, 39 years old, Mile)*

*Yes, they respected me, they told me about my general condition, the baby's position, and the potential hours of labor ... They took the time to explain to me right up until my delivery. They say only comforting and gentle words. (Woman, 31 years old, Kounoungou)*

The majority of providers reiterated the need, and the importance, of explaining the care they provide to the patient, and allowing the patient to agree or refuse. They also described explaining how the patient may feel during labor and delivery, especially for first-time mothers.

*Everything I do, I explain. Everything I do, I explain before I do it. If she says no, I can't force her....You explain everything you do as you go along. (Chadian midwife)*

*Respectful care during delivery. First, we have to help the patient get settled. You have to talk with the patient … You explain to her what is going to happen during labor. You explain to her how she will feel, that she’ll feel pain during this time. You have to explain it to her. So you're going to say when there's a contraction, she has to push. And if there's no contraction, she has to breathe. (Chadian midwife)*

Similarly, women, when prompted, reported that providers were clear with their explanations of the care they were going to provide and asked for consent. Only one woman mentioned being given an injection without first being informed, and they only explained to her after it was already administered. This was the only instance of non-consented care reported.

*Yes, she injected me without asking my opinion. And when I asked about it, she told me that you have difficulties in childbirth, that's why I have given you this injection to help you a little. ... Me, I thought that as she didn’t like me too much, maybe she was going to inject me and I die. (Woman, 20 years old, Mile)*

### Language barriers

While most women described the staff as encouraging them and providing guidance, a few mentioned being uncomfortable or afraid to ask questions. In addition, a number of women commented on language barriers between some of the Chadian midwives and Sudanese patients, describing the lack of translators as negatively impacting communication.

*Some patients speak in Arabic, in Zaghawa... and especially the people from the village do not know how to speak Arabic…. Sometimes there are too many disputes with the patients, the midwives don't listen well and they get very angry which is not good during the [ANC] consultation. Before, there were 3 translators for Zaghawa, Tama, and Arabic but now there are none. …You have to be nice to pregnant women because they are too frail. When there are no translators in the three languages, it’s complicated. (Woman, 27 years old, Mile)*

## Privacy/confidentiality

Most women described the delivery room as offering sufficient privacy, and nearly all reported that no one except the midwives could see them during their delivery. Although few women discussed confidentiality, those that did believed the providers kept their conversations private. Providers discussed the importance of maintaining confidentiality, especially with respect to adolescents who faced community stigma and discrimination in seeking care. For example, one midwife reported that unmarried girls are often deterred from visiting the maternity because they might be seen by other women waiting for care. Another talked about reassuring young clients that everything they discussed would remain between them and would not be discussed with anyone else. Providers also mentioned assisting adolescent girls to access services confidentially, by encouraging them to come early or late when fewer adult patients would be around to see them.

*It's like I just explained to you there. If you are not married and you come here, people think that you are pregnant, or that you were raped. But when you are married, they think you came because of pregnancy or maybe you came for any other visit. ... Sometimes even for the married ones, when they [adolescents] come and see many women here, they are afraid to enter. Sometimes they come to the window and whistle at me. So I go out the back and bring them directly into the room to serve them too. (Chadian midwife)*

## Social support

Although women typically arrived at the maternity with family members who supported them during labor and post-partum, birth companions were not permitted in the delivery room. This was a cause of anxiety and unease for a few women, although most recognized the reasons why (limited space and overcrowding) and were comfortable with the midwives and refugee providers. One woman reported that her aunt was permitted to enter and assist in the delivery room because the midwife was working alone.

## Health facility environment

Overall, women described the maternity as clean and well set up with sufficient privacy where the curtains, doors, or windows were closed.

*What I liked about the delivery room: the cleanliness, the hygiene, their [providers] greetings and communication with the patients. The room was well equipped with water too. Everyone likes cleanliness in life. (Woman, 28 years old, Mile)*

However, they also mentioned health system elements beyond the health workers’ control, including complaints about the physical space, supplies, and transportation, that affected their labor and delivery experience. The primary challenges to providing PCMC described by the providers also involved these health facility issues, such as feeling overworked, insufficient staff for the number of patients being seen daily at the maternity as well as issues with compensation (particularly among the refugee providers).

*The difficulties are many, such as [enough] chairs for us midwives to sit and rest. It’s been almost 20 years, and no change in materials. There are not enough beds for the patients. (Sudanese provider)*

*If the number of patients exceeds 4 to 5, some will sleep on the floor due to the lack of beds. We have only 3 [beds] in [the delivery room], and 2 [in the labor room] is very insufficient. If 6 to 8 patients come at the same time, some will stay on the floor*.  *(Sudanese provider)*

Both women and providers reported insufficient supplies and equipment, most notably beds, and occasionally soap and water in and around the maternity. Women expressed frustration at medication stockouts during ANC visits, especially when they were advised to purchase the medicines themselves at the pharmacy if they could afford it. Some women expressed disappointment with pain management after delivery, when they were only offered paracetamol. In addition, women were unhappy that the health facility no longer provided them with mama kits, such as baby wraps, clothing and soap. Women and providers in both camps described the limited beds and overcrowding as a major fault in the facility, and one woman even described it as a reason why some women don’t want to give birth at the maternities. When many patients came at once and the labor and delivery rooms were full, women in labor had to wait outside, sometimes sitting on the ground.

*When I arrived at the hospital, there were 5 other women also there for delivery. And the maternity ward has only 3 delivery beds...Three women found beds, one woman delivered on the floor, and the other two women waited outside, and in full view of everyone...When you ask the midwives, they tell you that the ward is full, and you sleep outside. (Woman, 37 years old, Kounoungou)*

In both camps, refugee providers reported the need for additional providers on staff to accommodate the volume of patients. They attributed challenges to providing PCMC, including anger expressed at patients, to overwork and stress. Despite the overwork, overall both the midwives and refugee providers described good collaboration and mutual support among the maternity staff.

*It's the result of stress, sometimes there's a lot of work and not enough staff at the center, so we end up getting angry. For example, we are only 6 [refugee] midwives in total in this center. We take turns being on duty, two by two. Sometimes there are a lot of pregnant women who come at the same time. So we end up collapsing under the weight of the work. (Sudanese providers)*

### Facility assessment

Each maternity was staffed by three midwives and six refugee TBAs plus one refugee assistant midwife at Mile. Overnight, one midwife and two refugee providers were present or on call in each maternity. Only the midwives were authorized to provide medication. The midwives used the partograph to monitor labor and delivery and performed active management of the third stage of labor. In the previous year, the maternities conducted 50-90 deliveries per month in Mile and 80-100 in Kounoungou. Each maternity had 2 (Kounoungou) or 3 (Mile) delivery beds, and 2 or 3 beds in the labor/postnatal room. The labor and delivery rooms were clean and ready for the next patients at the time of the assessment. No payment was asked of women prior to treatment unless they experienced a stockout of a needed medication and had to ask the woman to purchase them at the pharmacy. While neither maternity had an ambulance on site, the midwives could call either IRC or the MOH District Hospital in Guereda to send an ambulance if needed. Referrals therefore took 1-2 hours given the time needed for the vehicle to travel to the camp from Guereda.

The necessary supplies for infection prevention were available in (or just next to) the delivery rooms in both camps, including a washing station, soap, antiseptics, gloves, disinfection solution, covered trash can and puncture-proof receptacle for needles. While the maternities were well-equipped, two signal functions for basic EmONC were not provided, notably the administration of parenteral uterotonics and vacuum delivery. Although they used oxytocin for active management of the third stage of labor, the midwives were encouraged to refer patients with prolonged labor to the hospital. The midwives lacked training and equipment for vacuum delivery. Both maternities had most of the necessary supplies and equipment to conduct normal deliveries and treat some obstetric complications. Kounoungou was stocked out of suture materials in the delivery room, though they were available in the health center storeroom. Mile was stocked out of magnesium sulfate for eclampsia, and lacked injectable metronidazole for postpartum sepsis but did have gentamicin and ampicillin. Both maternities were stocked out of paracetamol or ibuprofen in the delivery room, although Mile had ibuprofen in the pharmacy. Both maternities had the needed equipment and supplies to provide contraceptive pills, injectables, implants and intra-uterine devices (IUD). Both were stocked out of male condoms. While Mile had dedicated emergency contraceptive pill packets, in Kounoungou they cut up regular contraceptive pill packets to make the correct dosage when emergency contraception was requested.

## Discussion

To the best of our knowledge, this is one of few studies to explore refugee women’s perceptions of person-centered maternity care. Overall, the findings suggested that the care provided at the maternities serving the two refugee camps was often, but not always, person-centered, with providers and women expressing similar perspectives. PCMC was reflected in providers’ descriptions of respectful care as supportive and fundamental to the care they provided and women’s reports of generally feeling respected and well cared for during labor and delivery. However, resource constraints, including crowded health facilities and overworked staff, did result in some women feeling disrespected or neglected during their childbirth experience suggesting room for improvement.

Unlike in some studies in Sub-Saharan Africa and other humanitarian settings [27–29], the providers in this study described the importance of PCMC in their work and for the wellbeing and positive health outcomes of their patients – both refugees and host population. This could be due, in part, to IRC’s emphasis on counseling, mentoring and working with providers to maintain respectful care. Providers personally showed pride and satisfaction in their work and thus delivered better care when they felt that their work was of importance [30]. These feelings may contribute to improved health worker performance as seen elsewhere [31–33]**.**

Consent and good communication were perceived as hallmarks of respect by both providers and women. Effective communication between providers and clients–where the providers clearly explain the plan and women feel comfortable asking questions–is essential for PCMC [12, 34, 35], and was largely reported here. This is even more important in a refugee setting where the power differential between refugees and host country providers may be considerable. Despite reports of overall positive communication with providers, a few women described some communication difficulties, particularly with the Chadian midwives who had varying Arabic fluency and who did not speak local Darfur languages. Some women and refugee providers described a need for interpreters in the maternity for women who did not speak Arabic, an important recommendation given that language is a substantial barrier to effective provider-patient communication [34]. A lack of interpreters to facilitate the dialogue needed to establish rapport and patient confidence in provider’s actions, as well as ensure the woman can make informed decisions about her care, negatively affects service delivery and may contribute to discrimination [35]. While both providers and women interviewed for this study did not report discrimination based on culture or ethnic group, the availability of interpreters would reduce the potential for such behavior.

Confidentiality was another attribute that providers described as being fundamental to maintaining good relationships with patients. Although all participants in this study were 18 or older, providers discussed experiences where adolescents or unmarried women would visit the maternity in secret for maternity or other reproductive health services such as contraception. Stigma associated with non-marital or adolescent sexual activity can deter young women from accessing these services [36]. Adolescent girls and women in humanitarian settings already experience many barriers to receiving effective contraceptives as compared to those in stable settings [36, 37]. However, the Chadian midwives’ attitudes in this study were generally positive when it came to assisting adolescent girls to obtain services in secret, and viewed it as an important part of their role to provide care regardless of the patient’s circumstances, in contrast to findings elsewhere reporting provider bias against adolescents [38].

The support of a birth companion has been seen to have positive effects on birthing outcomes [39]. Birth companions offer emotional and often physical support during labor and delivery and can improve the birthing experience for women overall [40, 41]. Women in this study explained that while they were allowed to have someone accompany them to the maternity for support during labor and the post-partum period, their companion was not allowed in the delivery room with them, except in extraordinary cases. Few women mentioned this as a problem, perhaps recognizing the limited space and overcrowding in the delivery room at these maternities. It is also possible that in this setting, the TBAs, who were from the refugee population, fill the role of a birth companion for women given their presence throughout labor and delivery at the health center. Despite the recommendation that women have a birth companion of their choice throughout their pregnancy and ideally during delivery [40], our findings are consistent with a study in Kenya that found most women wanted a birth companion with them during labor and after delivery, but were less interested in having one during delivery [42].

While no women in our study reported physical abuse, several described incidents of scolding, particularly when the providers were very busy. While the midwives largely did not rationalize such mistreatment, in contrast to findings elsewhere [43, 44], both women’s and providers’ reports of such negative behavior from providers were often attributed to overwork or a lack of supplies at the maternities. These factors have been documented as contributing to mistreatment of women in other settings [7, 30], and may be exacerbated in humanitarian crises. In the facility assessments, we found that most of the supplies and medications and equipment needed for delivery were present the day of the assessment. However, the issue of medication stockouts during ANC often arose in interviews. Stockouts contribute to a negative and less efficient workplace: when needed materials are lacking, a provider’s job is made more difficult [30]. Although services are free in the maternities serving refugees in this setting, when stockouts occurred, women were sometimes asked to pay for medications at a pharmacy - this appeared to occur for ANC visits and was not reported for delivery care. Being asked to pay out of pocket reduces willingness and ability to seek and use care [45, 46]. Even if women only experienced this during ANC, it may be sufficient to discourage some from coming for delivery care where they may anticipate a similar experience. Many women expressed frustration with overcrowding, especially the lack of sufficient beds in the labor and delivery rooms or being made to wait for long periods of time unattended. Such neglect can be dangerous for the mother and infant, but also makes women feel disrespected and discourages them from delivering at the health facility [7, 43]. Further while these health systems issues may provide context for women’s experiences of disrespect, they should not allow dismissal of women’s feelings of mistreatment, despite the positive attitudes and intentions of the providers [7].

As found in other low resource settings, when such scarcity and overwork is normalized within an organization, providers adopt workarounds which may result in mistreating women in childbirth [30, 47]. Addressing this scarcity and affirming the value of respect and support as essential components of care at an organizational level is critical to reduce incidents of mistreatment and disrespect [47]. In general, IRC has prioritized respectful care in this setting through refresher trainings, supportive supervision, coaching and mentoring, which have also shown some success elsewhere [30]. Integrating the refugee providers into the maternity team may contribute to better care for women as they are from the community, and also fill a complementary role with training to support and reduce the burden on the midwives, similar to on-call birth companions piloted in Tanzania or community-based doulas for refugees in high income countries [48, 49]. However, refugee providers in particular mentioned feeling overworked while also seeing their compensation reduced resulting in their feeling undervalued, similar to findings elsewhere [30]. Reduced donor funding, particularly in protracted humanitarian settings, can increase burdens on providers as funding decreases while the number of people to serve remains constant or increases [50]. For example, according to United Nations Office for the Coordination of Humanitarian Affairs’ (UN OCHA) Financial Tracking System, the average annual amount of humanitarian health funding committed for Chad in 2027-2022 was less than 40% of the annual average in 2010-2016 [51]. The decreased funding was reflected in comments from both refugee providers (compensation) and women (mama kits and medications provided during ANC). To avoid normalization of negative behavior, it is critical for organizations to better support and empower providers to provide good quality care - even during humanitarian crises, where overwork and lack of resources are common. This also applies to the MOH which must play a key role in health system strengthening to improve PCMC, even in refugee camps like these, so that women can achieve their right to respectful maternity care [13].

### Limitations

Although the interviewers were not IRC staff, they were recruited by IRC, so the possibility of courtesy bias from participants must be considered. Interviewers repeatedly reminded respondents that all information they provided was confidential and anonymous, but participants may not have felt fully comfortable in voicing their displeasure with the care or support that was provided or the support (or lack thereof) that they were receiving from IRC or others in the maternities. All of the women that we interviewed were 18 years or older as the selected younger adolescent women who unavailable; thus, their perspectives and additional issues they may face are missing here. Finally, women talked a lot about their experiences during ANC visits, despite the interviewer guiding them back to talking about their labor and delivery experience. Although the researchers did their best to distinguish between comments about each service, this was not always possible, meaning that some negative comments may have been mistakenly attributed to the childbirth experience rather than ANC.

## Conclusion

This study, one of the few that explored PCMC in a refugee camp, suggests that while the care in this setting was better than in some contexts, ensuring adequate resources and support enable providers to effectively perform. The MOH must be fully engaged in these activities in recognition of their role in the health system and to strengthen their organizational commitment and capacity for PCMC. Despite providers’ commitment to offering person-centered care and women’s generally positive experiences, conflict and displacement exacerbates the conditions that contribute to mistreatment during labor and delivery. Organizational emphasis and support for PCMC can mitigate these factors so that refugee women receive good quality delivery care.

### Supplementary Information

**Supplementary Material 1.** 

**Supplementary Material 2.** 

## Data Availability

The data that support the findings of this study are available from the corresponding author on reasonable request.

## Abbreviations

ANC: Antenatal care
EmONC: Emergency obstetric and newborn care
HIV: Human immunodeficiency virus
IRC: International Rescue Committee
IUD: Intra-uterine device
MOH: Ministry of Health
PCMC: Person-centered maternity care
ProGeSan: Protection, Gender and Health program
TBA: Traditional birth attendant
WHO: World Health Organization’s

## Footnotes

1 The 7 signal functions of basic EmONC: administer parenteral antibiotics; administer uterotonic drugs; administer parenteral anticonvulsants for pre-eclampsia and eclampsia; manually remove the placenta; remove retained products of conception; perform assisted vaginal delivery; perform basic neonatal resuscitation. *(Monitoring Emergency Obstetric Care: A Handbook. Geneva: World Health Organization, 2009).*
