# Supplementary material for: Refugee women’s and providers’ perceptions of person-centered maternity care: a qualitative study in two refugee camps in Chad
Source: BMC Pregnancy Childbirth. 2024 Apr 1;24:225. doi: 10.1186/s12884-024-06424-z (PMC10983620; doi:10.1186/s12884-024-06424-z)
Supplement: Supplementary file 1 — Supplementary Material 1. [file 12884_2024_6424_MOESM1_ESM.docx]

**Additional file 1: In-depth interview guides**

**IDI guide for women who delivered recently in the health center**

**Socio-demographic info**

- How old are you?
- How many times have you been pregnant?
- How many children do you have?
- How old were you when you gave birth to your first child?
- Have you ever attended school? What is the highest grade of school you completed?
- How many times did you visit this health center during your pregnancy, before the delivery?

**I would like to learn about your experiences getting care at the health facility during your last delivery.** **And please remember that everything you tell me is confidential and will not be shared with staff at the health facility.**

1. **Think about your delivery experience (the day you delivered). Can you walk me through your appointment from the moment you arrived at the clinic until you left.**
   - How was your experience?
   - How did you feel at the health facility? (Probe: (dis)comfort, privacy, curtains, screens, others watching, overcrowding, etc.)
   - Who helped you during delivery? How did this person help you?
   - Probe throughout: *How did you feel provider handled that/treated you in that moment*?
2. **Please tell me about the people from the clinic/facility that you interacted with. Who were all the people who provided care to you at the clinic?**
   - What did each person do?
   - What kinds of things did you like about the staff at the clinic?
   - How were you treated by people at the clinic? (Probe: shame, respect, neglect, humiliation, physical abuse, etc.)
   - What did you not like about the staff at the clinic?
   - Probe: What happened? How did you feel?
3. **COMMUNICATION WITH PROVIDERS: Now I would like to talk to you about the way the midwives explained things to you.**
   - In general, how well did you understand what the midwife was doing during your appointments? (Probe: if services were explained, if she was confused)
   - Were you able to ask the midwife questions? Tell me more…
   - How did the midwife get permission from you to perform services? How did you feel about this?
   - Tell me about how the midwives/nurses/doctors spoke to you (supportive, encouraging, harsh/strict)? How did that make you feel?
4. **What makes you think a health facility has good quality services? What makes you think a health facility has bad quality services?**
5. **What actions, attitudes, or behaviors by a health worker would you consider disrespectful or abusive?**
6. **Do you think it’s important for a health worker to behave respectfully towards you? Why?**
   - What do you think you are entitled to?
7. **During your stay at the health facility for childbirth, did you ever see a health worker behave towards another woman in a way that you thought was disrespectful or abusive?**

IF YES, PROBE:

- - What did you see happen?
  - How did it make you feel when you saw that?
  - What, if any, were the consequences for her or her baby’s health?
  - What, if anything, did you do in response? Why or why not?

1. **Have you ever heard about a woman who has experienced disrespect or abuse during delivery at the health facility?**

IF YES, PROBE:

- - What did you hear happened?
  - How did it make you feel when you heard about it?
  - What, if any, were the consequences for her or her baby’s health?
  - What, if anything, did she or her family do in response?

1. **During your stay at the health facility for childbirth, did the health workers do or say anything that you thought was disrespectful or abusive?**

IF YES, PROBE:

- - What happened?
  - How did it make you feel?
  - What, if any, were the consequences for you or your baby’s health?
  - Why do you think the health worker behaved or acted in this way towards you?
  - Who did you tell about it? Why that person(s) or why no one?
  - What, if anything, did you or your family do in response? Why or why not? (If nothing was done, PROBE: What do you wish you could have done about it?)

**IDI guide for Maternity staff (midwives, Traditional Birth Attendants)**

# Section 1: Background on the respondent

1. I would like you tell me a little bit about yourself. What is your training (eg midwife, nurse, doctor, other)? In what year did you complete your training?
2. Please tell me about your work here in this health facility? How many years have you been in this position? Worked at this health facility?

# Section 2: How staff are treated

*We want to understand what makes you feel respected at work and what you feel you need in order to do your job. (Please remind participant that all responses will be confidential and their responses will not affect their job in any way).*

1. What is the most rewarding part of your job? Why?
2. What is the most demanding part of your job? Why?
3. Do you feel valued at work? Please explain.
   1. How would you describe your relationship to colleagues?
   2. And with your supervisor?
4. Now I would like you to tell me what makes you feel respected at work?

# Section 3: Definition of respectful care and its practice

1. What does respectful care during childbirth mean to you, as midwife?
   1. To what extent do the women who give birth in this health center receive the kind of 'respectful care' you have described?
2. In your opinion, how should women be treated when they come to the health center?
   1. To what extent does this health center offer the care that they consider respectable?

# Section 4: Women’s experience of mistreatment during childbirth

1. Have you ever in your career observed disrespect and abuse towards women in the maternity?
   1. Can you tell me what happened?
   2. In your opinion, how frequently does this situation occur? Describe.
   3. How did it make you feel to see this kind of situation?
   4. Why do you think behavior like this happens?

# Section 5: Factors that influence treatment during childbirth

1. In your opinion, what factors could influence the degree of respect that women receive in this health center?

PROBE the following if not mentioned:

- 1. Health center resources or infrastructure (beds, medication, equipment, overcrowding)
  2. Staff workload
  3. The number of staff
  4. The administration
  5. Other factors

1. Have you seen that some women are treated better than others at the health center? Please describe.
   1. If yes: What factors could be related?

Probe for issues related to patient’s age, ethnicity, marital status, income, etc

1. In your opinion, what can be done to address these factors so that women are treated with respect during labor and childbirth?
2. What can we do to help you and other maternity staff to do your job?
3. Is there anything else that you would like to tell me?

**We are also interested in learning about adolescents’ access and use of sexual and reproductive health services. When we talk about adolescents, we mean girls and boys aged 12-19 years old. We’d like to hear your opinions about providing reproductive health services to adolescents at this facility.**

1. What would you say are the health needs of the adolescents in this community? How easy or difficult is it for the adolescents to discuss or talk about sexual and reproductive health?
2. What services do adolescents seek most often? Which SRH services?
3. What are your views on the utilization of SRH services among adolescents?
   1. What are some of the enabling factors in adolescents accessing SRH services ? What makes it easier for adolescents to receive services?
   2. What are some of the barriers that girls and boys face in accessing services in the facility? What makes it difficult for them to receive services?
   3. Do some groups of adolescents have more difficulty accessing services?

*Probe:* those who are less than a certain age; those who are not married; or those who belong to a certain group, such as people living or working on the street?

1. Are there some groups of adolescents who you do not feel comfortable treating?

*Probe* those less than a certain age, those who are unmarried

- 1. If so, could you explain why you feel uncomfortable?

1. Do community members support the provision of reproductive health services to adolescents?
   1. If so, do they assist you in any way? Explain how.
   2. Do they make it difficult to provide services? How?
   3. What do refugee health workers at the health facility think about provision of SRH services to adolescents?
2. In your opinion as a health worker, what can be done to improve adolescents’ access to and use of SRH services at the camp health center?
   1. What changes to clinic procedures could be most impactful?
   2. What else can be done through engagement with the families? Schools? Communities?
   3. What could help you and other health workers to provide better SRH services to adolescents?

***Thank you for your time. And I just want to reiterate that the information you shared will be kept private.***
