# Supplementary material for: Refugee women’s and providers’ perceptions of person-centered maternity care: a qualitative study in two refugee camps in Chad
Source: BMC Pregnancy Childbirth. 2024 Apr 1;24:225. doi: 10.1186/s12884-024-06424-z (PMC10983620; doi:10.1186/s12884-024-06424-z)
Supplement: Supplementary file 2 — Supplementary Material 2. [file 12884_2024_6424_MOESM2_ESM.docx]

**Supplementary information 2:** **Person-centered maternity care (PCMC) domains and codes**

*Separate codebooks for interviews with women and providers were developed. Most codes had positive and negative sub-codes.*

| **PCMC Domain** | **Codes - Women** | **Codes - Providers** |
| --- | --- | --- |
| Dignity and supportive care | - Welcome at the health facility - Providers’ behavior/tone - Woman’s comfort or general feeling | - Interpersonal relations with clients - Definition of respectful care - Description of satisfying and demanding aspects of her work - Discrimination |
| Communication and autonomy | Communication with the providers | - Description of midwife’s work - Definition of respectful care - Discrimination |
| Privacy and confidentiality | - Health facility environment - Communication with the providers | - Confidentiality - Discrimination |
| Social support | Accompaniment |  |
| Health facility environment | - Materials - Personnel - Health facility environment - Access - Referral | - Materials - Personnel |
| *Cross-cutting codes (often double coded with others)* | - Disrespect she heard about, observed or experienced - Reasons for respect or disrespect - Recommendations | - Examples of disrespect seen in this facility or elsewhere - Causes of disrespect - Interpersonal relations with colleagues - Recommendations |
